# An Atypical, Staged Cell Death Pathway Induced by Depletion of SNARE-Proteins MUNC18-1 or Syntaxin-1

**DOI:** 10.1523/JNEUROSCI.0611-22.2022

**Published:** 2023-01-18

**Authors:** Femke M. Feringa, Annemiek A. van Berkel, Anushka Nair, Matthijs Verhage

**Affiliations:** ^1^Deptartment of Functional Genomics, Center for Neurogenomics and Cognitive Research, VU University Amsterdam, 1081 HV Amsterdam, The Netherlands; ^2^Department of Human Genetics, Amsterdam University Medical Center, 1081 HV Amsterdam, The Netherlands

**Keywords:** cell death, Munc-18, SNARE, syntaxin

## Abstract

The presynaptic proteins MUNC18-1, syntaxin-1, and SNAP25 drive SNARE-mediated synaptic vesicle fusion and are also required for neuronal viability. Their absence triggers rapid, cell-autonomous, neuron-specific degeneration, unrelated to synaptic vesicle deficits. The underlying cell death pathways remain poorly understood. Here, we show that hippocampi of *munc18-1* null mice (unknown sex) express apoptosis hallmarks cleaved caspase 3 (CC-3) and phosphorylated p53, and have condensed nuclei. However, side-by-side *in vitro* comparison with classical apoptosis induced by camptothecin uncovered striking differences to syntaxin-1 and MUNC18-1 depleted neurons. First, live-cell imaging revealed consecutive neurite retraction hours before cell death in MUNC18-1 or syntaxin-1 depleted neurons, whereas all neurites retracted at once, directly before cell death in classical apoptosis. Second, CC-3 activation was observed only after loss of all neurites and cellular breakdown, whereas CC-3 is activated before any neurite loss in classical apoptosis. Third, a pan-caspase inhibitor and a p53 inhibitor both arrested classical apoptosis, as expected, but not cell death in MUNC18-1 or syntaxin-1 depleted neurons. Neuron-specific cell death, consecutive neurite retraction, and late CC-3 activation were conserved in syntaxin-1 depleted human neurons. Finally, no indications were observed for involvement of other established cell death pathways, including necroptosis, Wallerian degeneration, autophagic cell death, and pyroptosis. Together, these data show that depletion of presynaptic proteins MUNC18-1 or syntaxin-1 triggers an atypical, staged cell death pathway characterized by consecutive neurite retraction, ultimately leading to, but not driven by, apoptosis.

**SIGNIFICANCE STATEMENT** Neuronal cell death can occur via a multitude of pathways and plays an important role in the developing nervous system as well as neurodegenerative diseases. One poorly understood pathway to neuronal cell death takes place on depletion of presynaptic SNARE proteins syntaxin-1, SNAP25, or MUNC18-1. The current study demonstrates that MUNC18-1 or syntaxin-1 depleted neurons show a new, atypical, staged cell death that does not resemble any of the established cell death pathways in neurons. Cell death on MUNC18-1 or syntaxin-1 depletion is characterized by consecutive neurite retraction, ultimately involving, but not driven by, classical apoptosis.

## Introduction

During development, programmed cell death of neurons is crucial for the formation of functional circuitries (Kuan et al., 2000). Moreover, neuronal cell death is central to the pathogenesis of many brain diseases such as Alzheimer's or Parkinson's. It has now been well established that different types of neuronal cell death pathways exist, such as apoptosis or extrinsically driven necroptosis, which follow a defined cascade of events ultimately leading to regulated death (Fricker et al., 2018). However, upstream initiators of cell death and decision points for which cascade is initiated remain to be elucidated.

One poorly understood pathway is neuronal cell death initiated by depletion of presynaptic SNARE proteins syntaxin-1 (stx-1), SNAP25, or MUNC18-1 (M18-1). Depletion of these proteins leads to massive, cell-autonomous, neuron-specific degeneration (Heeroma et al., 2004; Peng et al., 2013; Santos et al., 2017; Vardar et al., 2016; Verhage et al., 2000; Berliocchi et al., 2005; van Berkel et al., 2021). The role of these proteins in viability is independent from their well-characterized function in synaptic transmission. Neuronal viability, but not synaptic transmission, is rescued on expression of non-neuronal paralogs (Delgado-Martínez et al., 2007; Peng et al., 2013; Santos et al., 2017). Conversely, depletion of several other presynaptic proteins, such as MUNC13-1, blocks synaptic transmission without affecting viability (Schoch et al., 2001; Varoqueaux et al., 2002).

The cellular mechanisms driving cell death on depletion of stx-1, SNAP25, or M18-1 remain unresolved. Initial reports showed that M18-1 null brains contain nuclei with compacted chromatin positive in TUNEL staining, indicating involvement of the apoptotic pathway (Verhage et al., 2000). This was further supported by increased cleaved caspase-3 (CC-3) reactivity in M18-1 null motor neurons (Law et al., 2016). Nuclear condensation and CC-3 reactivity was also observed in stx-1 depleted neurons (Peng et al., 2013; Berliocchi et al., 2005). However, genetic ablation of key apoptosis mediator APAF-1, sufficient to stop apoptosis-driven cell death, did not rescue viability in M18-1 null neurons (Honarpour et al., 2001). This suggests the involvement of other cell death pathways.

In the present study, we investigated the cell death pathway on depletion of M18-1 or stx-1 and compared it side by side with classical apoptosis. We confirm increased CC-3 reactivity in null neurons. However, live-cell imaging of neurons during the last hours before cell death revealed striking differences with the established apoptosis pathway, including consecutive neurite retraction and late CC-3 activation, only after complete neurite loss. Established apoptosis inhibitors were not effective in rescuing viability in M18-1 null neurons. No evidence was found for involvement of other well-known cell death pathways, such as necroptosis, pyroptosis, and parthanatos. Together, these data show that loss of M18-1 or stx-1 triggers an atypical, staged cell death pathway characterized by consecutive neurite retraction, ultimately leading to, but not driven by, apoptosis.

## Materials and Methods

### Animals

*Stxbp1* null (hereafter referred to as M18-1 null) mice were generated as described before (Verhage et al., 2000). In short, exons 2–6 were replaced with a neomycin resistance gene by homologous recombination, resulting in complete depletion of M18-1 expression. As depletion of M18-1 is lethal on birth, null mice were generated by crossing heterozygous mice. On embryonic day (E) 18 of pregnancy, mice were killed, and pups were obtained by caesarian section. Animals were housed and bred according to institutional, Dutch, and U.S. governmental guidelines.

### Neuronal cultures

Cortices were obtained from E 18 wild-type (WT) and M18-1 null embryos (unknown sex) and collected in HBSS (Sigma-Aldrich) containing 7 mm HEPES (Invitrogen). Tissue was incubated in HBSS-HEPES with 0.25% trypsin (Invitrogen) for 20 min at 37 C. After three washes, cortices were triturated with fire-polished Pasteur pipettes, and neurons were counted in a Fuchs–Rosenthal chamber. Neurons were plated on a layer of pregrown rat glia on 18 mm glass coverslips in prewarmed Neurobasal Medium (Invitrogen) supplemented with 2% B27 (Invitrogen), 1.8% HEPES, 0.25% GlutaMAX (Invitrogen), and 0.1% Penicillin-Streptomycin (Pen/Strep; Invitrogen). The glial feeder layer was created by plating rat glia on etched coverslips treated with 0.1 mg/ml poly-d-lysine and 0.2 mg/ml rat tail collagen (BD Biosciences). For monoculture, WT neurons were plated on 18 mm poly-l-ornithine/laminin-coated (Sigma-Aldrich) glass coverslips. WT and M18-1 null neurons were plated at a density of 25 K/well and 75 K/well, respectively.

To generate human neurons, the Bioini010-C-13 induced pluripotent stem cell (iPSC) line (biosample ID SAMEA103988285) was used, which has the NGN2 gene integrated in the AAVS1 safe harbor locus. iPSCs were maintained in Essential 8 (E8) medium (Invitrogen) supplemented with 0.1% Pen/Strep (Life Technologies) on Matrigel-coated plates. Cells were passaged using Gentle Cell Dissociation Reagent (StemCell Technologies), and on replating, E8 medium was supplemented with 5 um ROCK inhibitor Y27632 (Tetubio). To induce neurons, cells were grown in N2 medium (DMEM/f12 medium, Life Technologies), 200 mm GlutaMAX (Life Technologies), 20% Dextrose (Life Technologies), 1% N2 supplement B (StemCell Technologies), and 0.1% Pen/Strep) supplemented with 2 μg/ml doxycycline hyclate (Sigma-Aldrich) and dual SMAD inhibitors, 100 nm LDN193189 (Stemgent), 10 um SB431542 (Tocris Bioscience), and 2 um XAV939 (Stemgent). The medium was refreshed after 24 h. The next day, the medium was refreshed excluding the dual SMAD inhibitors but including 10 um to stop cell proliferation. The following day, neurons were replated on a layer of pregrown rat glia on 18 mm coverslips at a density of 200 K/well in neuronal maintenance medium (Neurobasal Medium supplemented with 200 mm GlutaMAX, 20% dextrose, nonessential amino acids (Life Technologies), B27, 0.1% Pen/Strep, 0.5% fetal bovine serum (Life Technologies), 10 ng/ml BDNF (PeproTech), 10 ng/ml CNTF (StemCell Technologies), 10 ng/ml GDNF (PeproTech), and 2 μg/ml doxycycline hyclate). Then medium was replaced with 50% fresh medium twice a week.

### Experimental design

A construct encoding pSyn(pr)EGFPLL3.7 was subcloned into lentiviral vectors, and viral particles were produced as described before (Naldini et al., 1996). WT and M18-1 null neurons were infected with lentiviral particles at 0 d *in vitro* (DIV). To induce classical apoptosis, neurons were treated with 10 um camptothecin (CPT; catalog #ALX-350-015, Enzo Life Sciences) or DMSO (catalog #D2438, Sigma-Aldrich) as control at DIV 3 and fixed 24 h later, or live-cell imaging was started 5 h after CPT addition at DIV 3. M18-1 null neurons were treated with Z-VAD-FMK (50 um, catalog #S7023, BioConnect), pifithrin-μ (10 um, catalog #P0122, Merck Millipore), or DMSO as control at DIV 2 and fixed at DIV 3 or used for live-cell imaging at DIV 3. To study Stx-1 loss, neurons were transfected with botulinum neurotoxin C (BoNT/C)-mCherry or mCherry(control) RNA using lipofectamine (Life Technologies). In short, cells were incubated in plain Neurobasal Medium with 10 mm MgCl_2_ for 1 h at 37C° followed by lipofectamine transfection performed according to the manufacturer protocol using 0.5 ug BoNT/C-mCherry RNA per 25,000 neurons in a 12-well plate. The medium was replaced for supplemented Neurobasal Medium 2 h after transfection, and cells were used for live-cell imaging 4 h after transfection or fixed 5 h after transfection. Cells were fixed at indicated time points after transfection (see Fig. 3*E*). Pifithrin-μ inhibitor was added from the start of RNA transfection (Fig. 3*E*). For live-cell monitoring of caspase 3 cleavage, neurons were treated with 1× NucView 488 (Biotium) 30 min before live-cell imaging.

### Immunocytochemistry

Whole brains isolated from E 18 mice were fixed with 4% paraformaldehyde (catalog #p6148, Sigma-Aldrich) in PBS for 72 h at 4C°. For gelatin embedding, brains were submerged in 30% sucrose (catalog #35579.02, SERVA) in PBS at least 24 h at 4C°. When brains sank to the bottom of the tube, the sucrose solution was replaced with double-distilled H_2_O (ddH_2_O), and brains in ddH_2_O were kept at 55C° for 45 min. The ddH_2_O was replaced with a 7% gelatin (catalog #9000-70-8, Merck Millipore) and 5% sucrose (catalog #A3935, AppliChem) solution in ddH_2_O, and brains were incubated for 2 h at 55C°, followed by 3 h incubation at 55C° in a 14% gelatin/10% sucrose solution. Brains in 14% gelatin solution were poured into small weigh boats on ice and positioned until the gelatin settled, followed by storage at 4C° for 1.5 h. Casted brains were cut out with a razor blade and stored in a 4% PFA/10% sucrose solution overnight at 4C°. Gelatin-embedded brains were washed with 0.5× PBS and stored in 30% sucrose solution in 0.5% PBS for at least 48 h at 4C°. To section brains into slices, casted brains were embedded in optimal cutting temperature compound Cryostat Embedding Medium (Scigen 4586). Slices of 50 μm were generated on a cryostat microtome (Cryostar NX50, Epredia) at −1°C and stored in PBS. Slices were treated with 3% H_2_O_2_ in 0.5× PBS 30 min at room temperature (RT) followed by 2 times PBS wash. Slices were incubated in blocking solution (3% BSA, 0.2% Triton X-100 in 1× PBS) for 1 h at RT, before incubation with primary antibodies in blocking solution for 48 h at 4C°. The following primary antibodies were used: mouse anti-phospho-p53ser15 (1:500; catalog #9286, Cell Signaling Technology), rabbit anti-cleaved caspase 3 (1:500; catalog #9661S; Bioké), mouse anti-p53 (DO1; catalog #sc-126, Santa Cruz Biotechnology) and rabbit anti-pMLKL (1:200; catalog #ab196436, Abcam). After 3 times 5 min wash in 1× PBS at RT, slices were incubated with secondary antibodies Alexa Fluor (1:1000; Invitrogen) in blocking solution for 4 h at RT combined with DAPI. Slices were washed three times 5 min in 1× PBS at RT and mounted on gelatin-coated microscopic slides with Mowiol-DABCO covered by a glass slide.

Neuronal cultures were fixed with 3.7% formaldehyde (formaldehyde reagent, catalog #15681, Electron Microscopy Sciences). After three washes with PBS, pH 7.4, neurons were permeabilized with 0.5% Triton X-100. Next, neurons were incubated for 30 min in PBS containing 0.1% Triton X-100 and 2% normal goat serum to block a-specific binding. Neurons were stained with primary antibodies, diluted in blocking solution, for 2 h at RT. The following primary antibodies were used: chicken anti-MAP2 (1:1500; catalog ab5392, Abcam; RRID:AB_2138153), rabbit anti-mCherry (1:1000; catalog #GTX128508, GeneTex; RRID:AB_2721247), mouse anti-LC3 (1:50; catalog #M152-3, MBL), rabbit anti-cleaved caspase 3 (1:1000; catalog #9661S, Bioké), and rabbit anti-pMLKL (1:200; catalog #ab196436, Abcam). Following three washes with PBS, neurons were stained with secondary antibodies Alexa Fluor (1:1000; Invitrogen) combined with DAPI for 1 h at RT. Neurons were again washed for 3 times in PBS, and coverslips were mounted on microscopic slides with Mowiol-DABCO.

**Figure 1.**
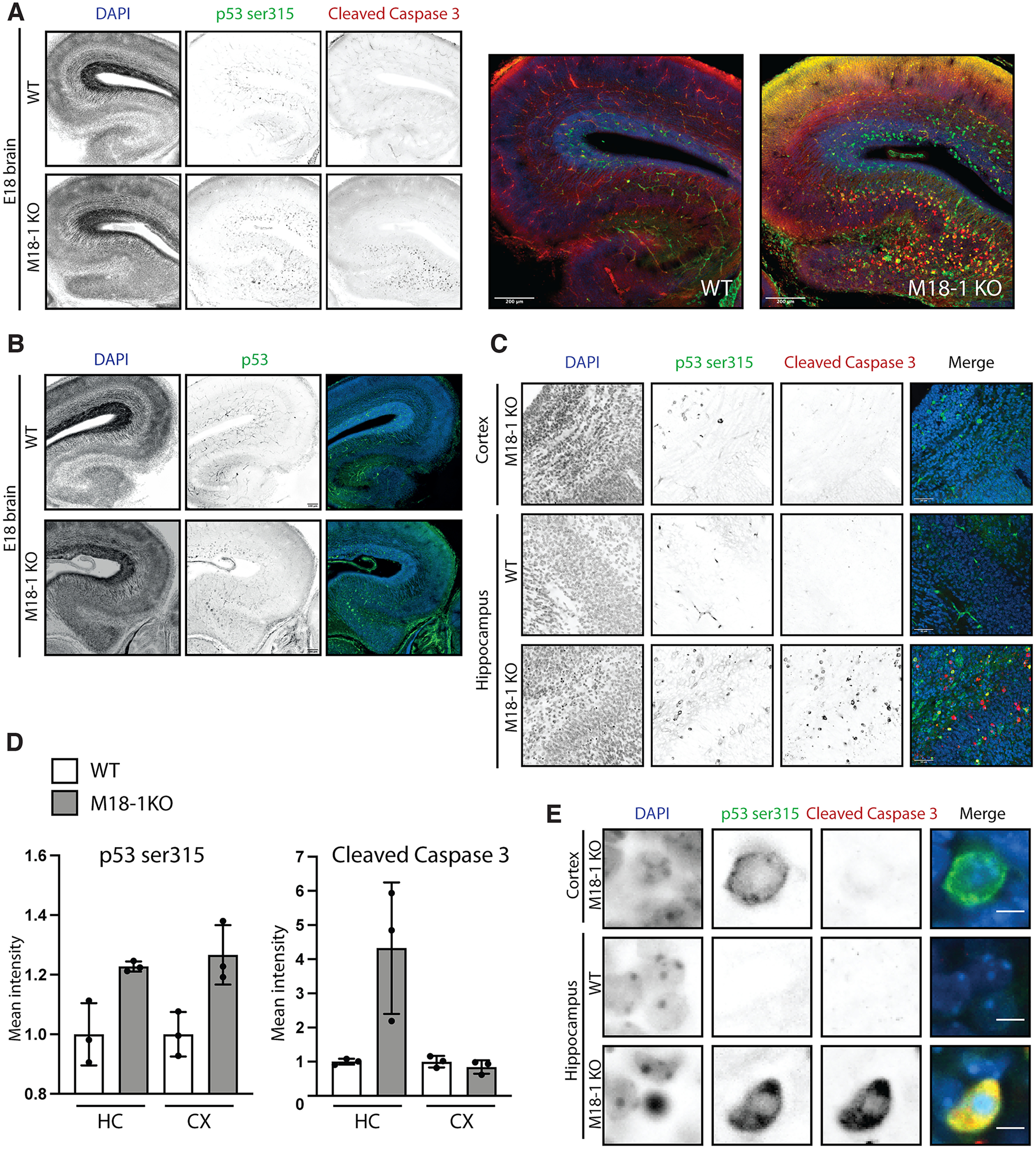
Progressive neurodegeneration in M18-1 null mice is characterized by phosphorylated P53 and cleaved caspase 3 activation. ***A***, Representative images of E 18 brain slices from WT or M18-1 null mice stained for p-p53 ser15 and CC-3. Scale bar, 200 μm. ***B***, Representative images of E 18 brain slices from WT or M18-1 null mice stained for total p53 protein. Scale bar, 100 μm. ***C***, Representative zoom of cortex or hippocampus from E 18 WT or M18-1 null brain slices. Scale bar, 50 μm. ***D***, Quantification of p-p53 ser15 and CC-3 signal in the indicated brain regions from a representative WT or M18-1 null brain. Signal intensities from three random rectangular regions in M18-1 null hippocampus or cortex were normalized to the mean signal intensity of three similar regions in the WT brain (excluding regions with obvious blood vessel staining). Mean ± SD. ***E***, Zoom of a single cell from cortex or hippocampus in WT and M18-1 null E 18 slices stained as in ***A***. Scale bar, 5 μm.

Images were made on a Nikon Ti-Eclipse microscope, equipped with a confocal scanner model A1R+, using a 40× oil immersion objective (NA = 1.3) or a 60× oil immersion objective (NA = 1.4) with 1× zoom. Confocal settings were optimized for every culture batch, and *Z*-stacks were acquired with 0.5 μm intervals. For analysis, *Z*-stacks were collapsed to maximal projections. For representative images of slices in Figure 1, the brightness and contrast values of p53 and DAPI staining were adjusted to represent equal background signals and distinguish the signal of interest for each example. Quantification of phospho p53 ser15 and CC-3 signal in hippocampus and cortex shown (Fig. 1*D*) is from one representative biological replicate and relative to the local background signal because of the high variety of background signal between imaged brain slices (in total, brain slices from three mice were analyzed). For neuronal density measures images were made on a Nikon Ti-Eclipse microscope, equipped with a confocal scanner model A1R+, using a 10× air objective (NA = 0.3) with 1× zoom. A minimum of 20 fields per condition were imaged for each biological replicate. The number of neurons with >2 neurites in each field were averaged to quantify neuronal survival. For the neurite number (see Fig. 3*E*), the mean number of neurites per soma for 8–21 cells per replicate was determined based on MAP2 staining in mCherry-positive cells.

### Live-cell imaging

DIV 3 neurons were transferred to a Nikon Ti-Eclipse microscope equipped with a confocal scanner model A1R+, 40× oil (NA = 1.3) objective and Tokai Hit system prewarmed to 37 C° with 5% CO_2_. Time-lapse recordings were made using resonant scanning mode. For morphologic analysis (see Fig. 3), image acquisition was performed every 15 min for a total of 16 h with 2× zoom and *Z*-stacks of 1 μm interval. For live CC-3 imaging (see Fig. 4), 1 μl/ml NucView 488 (Biotum) was added at *t* = 0, and image acquisition was performed every 7.5 min for a total of 4 h with one zoom and *Z*-stacks with 1 μm intervals. In both experiments, neurons were imaged in supplemented Neurobasal Medium.

### Statistical analysis

Statistical analysis and graphing were performed using GraphPad Prism version 9. No test for outliers was performed. Parametric tests were used whenever assumptions of homoscedasticity and normality were met. Otherwise, nonparametric tests were used when possible; *p* values below 0.05 were accepted as statistically significant.

## Results

### Abundant expression of phosphorylated P53 and cleaved caspase 3 in M18-1 null mice

To better define neuronal cell death in *munc18-1/*M18-1 null brains, E 18 brain slices were stained for apoptosis-marker CC-3 (Yuan and Yankner, 2000). Cleavage of caspase 3 marks the end stage of apoptotic cell death where cleaved caspase 3 is responsible for cellular destruction (Tewari et al., 1995; Liu et al., 1997; Kothakota et al., 1997). Hippocampi from M18-1 null mice presented large numbers of cells marked by CC-3, which were virtually absent in hippocampi from WT mice (Fig. 1*A*).

Apoptotic cell death is commonly preceded by activation of the cellular stress protein p53, which can be established by phosphorylation at serine 15 (p53 ser15; Hafner et al., 2019). Increased p53 ser15 staining was observed in hippocampi from M18-1 null mice, which overlapped only partially with CC-3-positive cells (Fig. 1*A*,*C*). In contrast to CC-3, phosphorylated p53-positive cells were also present abundantly in the cortex of E 18 M18-1 null, but not wild-type brains (Fig. 1*A*,*C*,*D*). A similar pattern was detected when slices were stained for total p53 levels (Fig. 1*B*). Absence of CC-3 staining in the cortex of M18-1 null mice suggests that cell death is not (yet) initiated in this brain region. Cells with condensed or fragmented nuclei, as detected by DAPI staining, were exclusively found in the hippocampi from M18-1 null mice (Fig. 1*E*). Hence, cell death in M18-1 null brains is characterized by increased levels of activated p53, CC-3, and condensed chromatin, suggesting activation of the apoptotic pathway.

### No indications for necroptosis, Wallerian degeneration, or autophagic cell death in M18-1 null neurons

**Figure 2.**
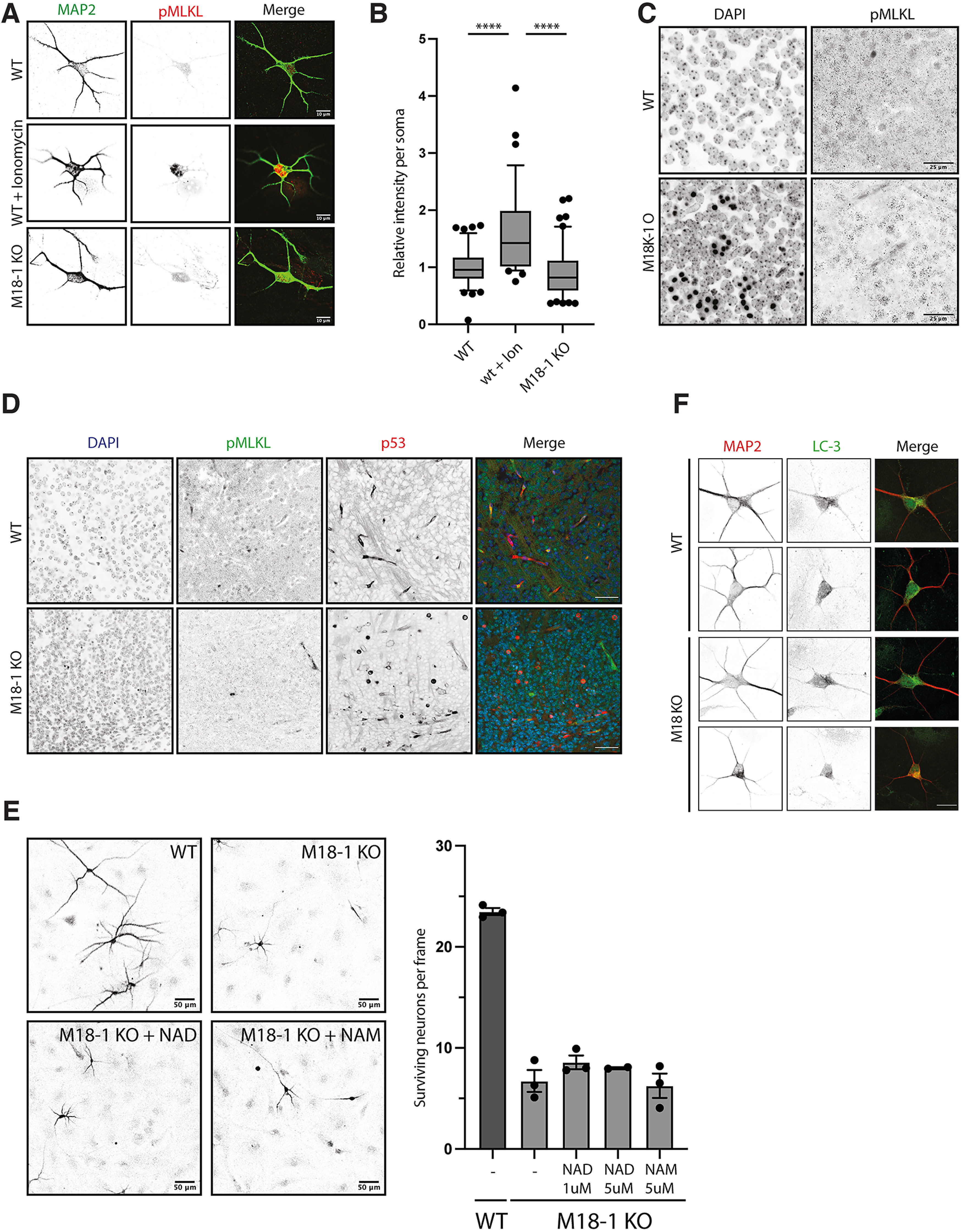
No indications for necroptosis, Wallerian degeneration, or autophagic cell death in M18-1 null neurons. ***A***, Typical examples of pMLKL staining in soma of WT neurons treated with Ionomycin at DIV 3 or M18-1 null neurons. ***B***, Quantification of pMLKL staining in soma as treated in ***A***. Box plot with error bars indicating 10th–90th percentile; *n* > 39 cells per condition from two biological replicates (*p* < 0.0001, 1-way ANOVA with Tukey's multiple comparisons test). ***C***, Representative images of E 18 brain slices in hippocampus from WT or M18-1 null mice stained for pMLKL. Scale bar, 25 μm. ***D***, Representative images of E 18 brain slices in hippocampus from WT or M18-1 null mice stained for pMLKL and p53. Scale bar, 200 μm. ***E***, Typical examples of DIV 3 WT neurons, M18-1 null neurons, or M18-1 null neurons treated with NAD (5 μm) or NAM (5 μm) for 24 h. Quantification of neuronal survival at DIV 3 of M18-1 null neurons and M18-1 null neurons treated with indicated concentrations of NAD or NAM for 24 h. Mean + SEM of three independent experiments. ***F***, Typical examples of LC-3 staining in WT neurons or M18-1 null neurons at DIV 3. Scale bar, 20 μm.

We also investigated the potential involvement of other cell death pathways, that is, necroptosis, Wallerian degeneration, and autophagy in M18-1 null neurons. Necroptosis is characterized by increased phosphorylation of mixed lineage kinase domain-like (pMLKL) and rupture of the plasma membrane and can be induced by external factors such as Ionomycin (Fricker et al., 2018; Gwag et al., 1999; González-Juarbe et al., 2017). WT neurons treated with Ionomycin indeed showed increased reactivity for pMLKL (Fig. 2*A*,*B*). In contrast, increased reactivity for pMLKL or membrane rupture was not observed in M18-1 null primary cortical cultures or brain slices (Fig. 2*A*–*D*). Axonal Wallerian degeneration is characterized by the fact that exogenous treatment of nicotinamide adenine dinucleotide (NAD) or the upstream precursor nicotinamide (NAM; Araki et al., 2004) rescues or delays degeneration. Treatment with NAD or NAM did not rescue or delay degeneration in M18-1 null neurons (Fig. 2*E*). Finally, activation of the autophagic pathway is characterized by LC3-positive puncta (Kabeya et al., 2000). We found no LC3-positive puncta in M18-1 null neurons (Fig. 2*F*). Together, no indications were observed for the involvement of necroptotic, Wallerian degeneration and autophagic cell death pathways in M18-1 null neurons.

### Atypical, staged cell death in M18-1/stx-1 depleted neurons

**Figure 3.**
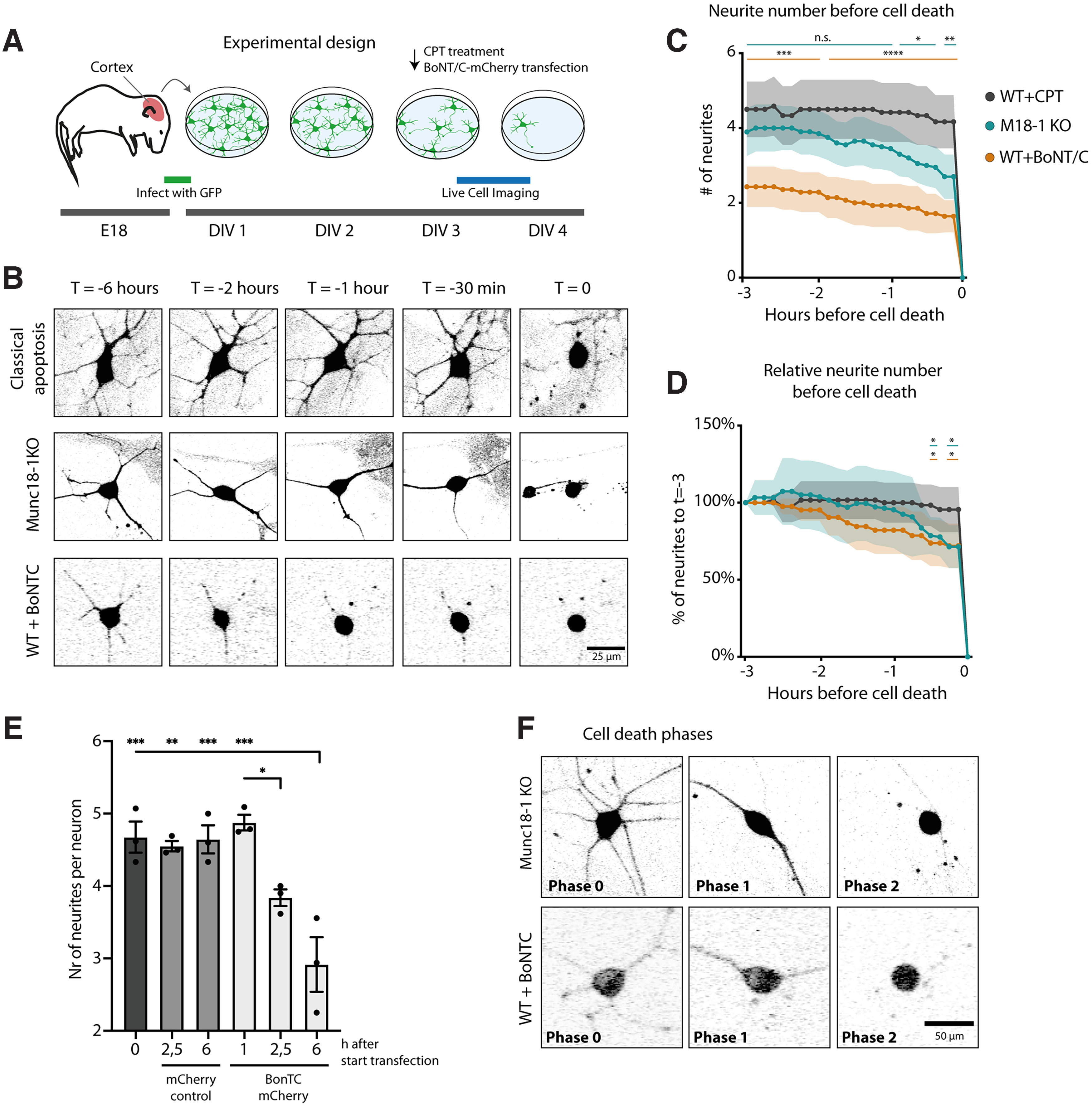
Morphologic stages before cell death in M18-1 null neurons and in WT neurons treated with BoNT/C or CPT. ***A***, Experimental design. E 18 cortical neurons were infected with GFP and cultured for 3 d. Then live-cell imaging was performed to visualize morphological stages before cell death. ***B***, Typical examples of WT neurons treated with CPT or BoNT/C and M18-1 null neurons in the hours before cell death. Scale bar, 25 μm. Movie 1, Live-cell imaging of WT cortical neurons treated with 10 um camptothecin at DIV 3 imaged at DIV 3–4. WT cortical neurons treated with CPT (classical apoptosis). Movie 2, Live-cell imaging of M18-1 KO cortical neurons imaged at DIV 3. M18-1 null neurons. Stills of movie show last timepoint before cell death. ***C***, Number of neurites were counted in neurons upon live-cell imaging 3 h before cell death. Mean + CI 95% (CPT, *n*/*N* = 12/3; M18-1 null, *n*/*N* = 20/3; BoNT/C, *n*/*N* = 14/3). Two-way ANOVA revealed a significant difference in average neurite number by both condition (*F*_(2,43)_ = 16.98, *p* < 0.0001) and time (*F*_(3.497,150.4)_ = 71.16, *p* < 0.0001) and interaction of both terms (*F*_(48,1032)_ = 3.3836). *Post hoc* Dunnett's multiple comparisons (corrected for multiple testing) revealed a significant difference in neurite number for all time points for stx-1 depleted neurons and the last hour for M18-1 null neurons; *p < 0.05, **p < 0.01, ***p < 0.001, ****p < 0.0001). ***D***, Neurite number of individual neurons relative to 3 h before cell death. Two-way ANOVA revealed a significant difference in average neurite number by time (*F*_(2.422,104.1)_ = 56.58, *p* < 0.0001) and interaction of time and condition (*F*_(48,1032)_ = 1.473, *p* = 0.02), but not by condition (*F*_(2,43)_ = 0.71, *p* > 0.05). *Post hoc* Dunnett's multiple comparisons (corrected for multiple testing) revealed a significant difference in relative neurite number of stx-1 and M18-1 depleted neurons in the 30 min before cell death compared with WT + CPT neurons; *p < 0.05, **p < 0.01, ***p < 0.001, ****p < 0.0001). Thirty minutes before cell death, M18-1 null and BoNT/C-treated neurons were significantly lower compared with CPT-treated neurons (CPT, *n*/*N* = 12/3; M18-1 null, *n*/*N* = 20/3; BoNT/C, *n*/*N* = 14/3). ***E***, Mean number (Nr) of neurites per neuron in mCherry-positive cells quantified at the indicated time points after RNA transfection. Mean + SEM of three independent experiments (*p < 0.05, **p < 0.01, ***p < 0.001; 1-way ANOVA with Tukey's multiple comparisons test). ***F***, Typical examples of the morphologic stages before cell death in M18-1 null and BoNT/C-treated neurons. Scale bar, 50 μm.

To further assess whether neuronal cell death on depletion of M18-1 or stx-1 follows the classical apoptotic pathway, different stages before cell death were compared with neurons treated with topoisomerase inhibitor CPT, a classical apoptosis inducer (Morris and Geller, 1996; Traganos et al., 1996). To this end, we performed live-cell imaging of primary cortical neurons from M18-1 null mice, BoNT/C-treated WT neurons to deplete stx-1, and CPT-treated WT neurons (Fig. 3*A*). The morphology of neurons treated with CPT remained unchanged until directly before cell death occurred (Fig. 3*B–D*; Movie 1). Multiple hours before cell death, M18-1 or stx-1 depleted neurons already had fewer neurites and branch points compared with WT neurons (Fig. 3*B*). Also, cell death in M18-1 or stx-1 depleted neurons was preceded by consecutive loss of neurites, which were retracted one by one (Fig. 3*B–E*; Movie 2). This resulted in a significant difference in absolute number of neurites by both condition and by time, as well as a significant interaction between these terms (Fig. 3*C*). *Post hoc* comparisons revealed that for stx-1 depleted neurons, the number of neurites was significantly lower for all time points, whereas for M18-1 null neurons this became significant 1 h before cell death (Fig. 3*C*). The progressive loss of neurites was further studied by examining the relative number of neurites compared with the number 3 h before cell death of individual neurons (Fig. 3*D*). For WT+CPT neurons, the relative number of neurites remained constant until cell death. For stx-1 and M18-1 depleted neurons, however, the relative number of neurites decreased over time and became significantly different from WT+CPT 30 min before cell death (Fig. 3*D*). The morphologic changes before cell death on M18-1 or stx-1 depletion can be characterized by different phases (Fig. 3*F*); in Phase 0, neurons are morphologically normal, then in Phase 1, neurites start to retract, leaving only one or two neurites. This phase takes hours on stx-1 depletion, whereas for M18-1 null neurons this is restricted to the last 2 h before cell death. Finally neurons die at Phase 2 (Fig. 3*F*). This is in contrast to CPT treatment, which does not affect neurite numbers before cell death, although we cannot exclude that such changes happen below our sampling resolution. Together, unlike typical apoptosis, M18-1 or stx-1 depletion leads to consecutive loss of neurites resulting in distinct morphologic phases hours before cell death.

Movie 1. Neuronal cell death after CPT treatment. A representative movie showing classical apoptosis of a DIV 3 primary neuron treated with CPT. The movie shows the neuron from 6 h until the moment of cell death. No apparent changes in neurite morphology are visible until the moment of cell death. 10.1523/JNEUROSCI.0611-22.2022.video.1

Movie 2. Neuronal cell death in a M18-1 KO neuron. A representative movie showing atypical cell death of a DIV 3 primary neuron knockout for M18-1. The movie shows the neuron from 6 h until the moment of cell death. The retraction of neurites one by one can be seen hours before actual cell death. 10.1523/JNEUROSCI.0611-22.2022.video.2

### Cleaved caspase 3 activation in the final phase of cell death in M18-1 null neurons

**Figure 4.**
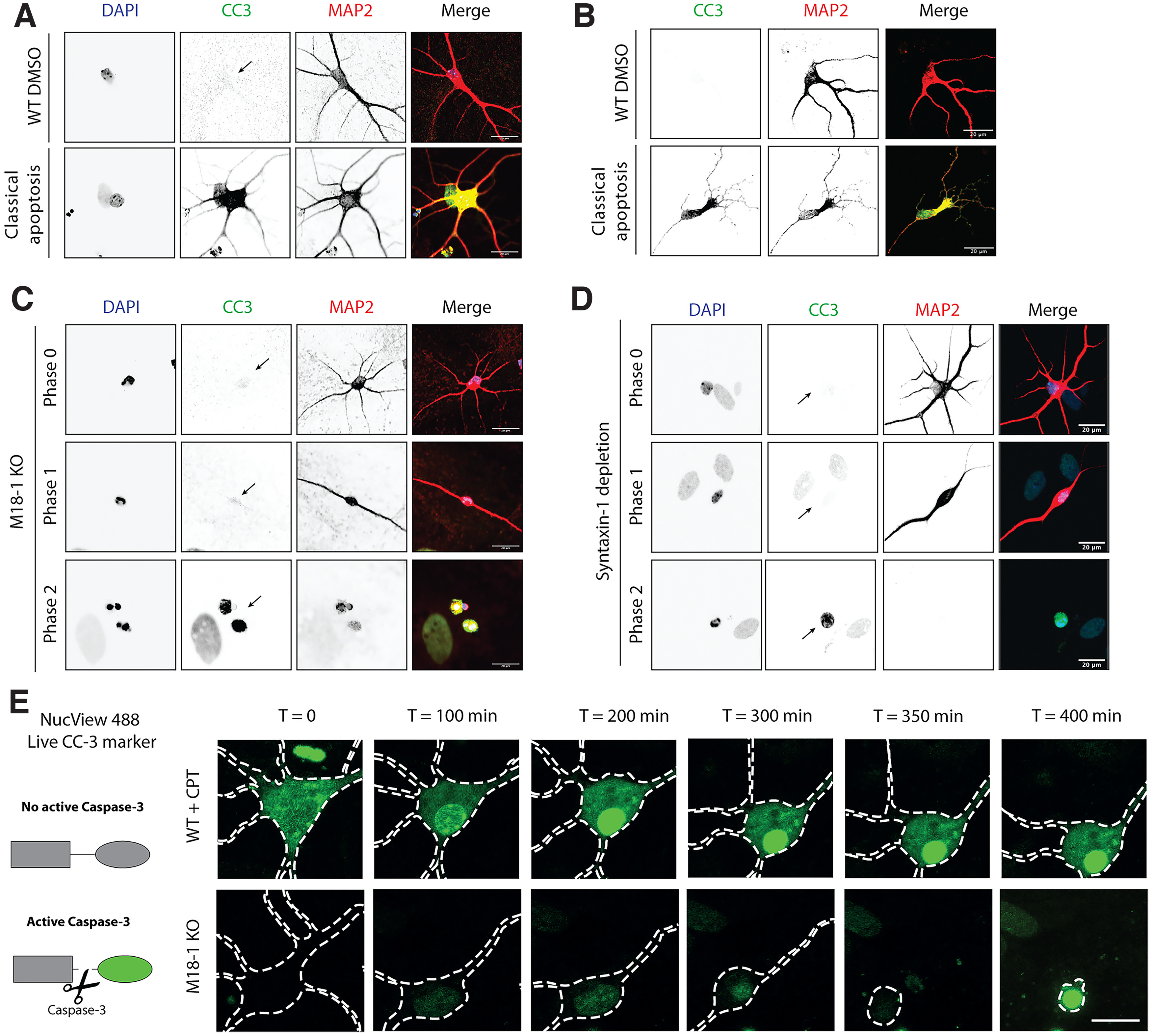
Differential cleaved caspase 3 activation in M18-1 null or BoNT/C-treated neurons compared with CPT-treated WT neurons. ***A***, Typical examples of CC-3 staining in WT neurons treated with CPT at DIV 3. Arrows (***A–D***) indicate location of the soma. ***B***, Typical examples of CC-3 staining in WT neurons cultured in absence of glia and treated with CPT for 4 h at DIV 3. ***C***, Typical examples of CC-3 staining in M18-1 null neurons at different phases of cell death. ***D***, Typical examples of CC-3 staining in WT neurons treated with BoNT/C at different phases of cell death. ***E***, Representative stills from WT neurons treated with CPT or M18-1 null neurons. Cells are treated with NucView 488 to track CC-3 activation by live-cell imaging. Scale bar, 50 μm.

Next, we investigated how activation of CC-3 relates to these distinct morphologic phases. In neurons undergoing typical apoptosis, as induced by CPT, pan-neuronal immunoreactivity for CC-3 was observed while neuronal morphology was still intact (Fig. 4*A*). This response did not depend on the glial layer supporting the primary neurons as the pan-neuronal CC-3 activation was also observed in CPT-treated neurons cultured in absence of glia (Fig. 4*B*). In contrast, CC-3 staining was not detected in the initial phases that preceded cell death in M18-1 null neurons (Fig. 4*C*) or Stx-1 depleted neurons (Fig. 4*D*). CC-3 only appeared in phase 2 neurons, when all neurites and associated MAP2 staining was lost (Fig. 4*C*,*D*). Similar observations were made when CPT-treated and M18-1 null neurons were tracked by real-time imaging with a live-cell marker for CC-3 activation (Fig. 4*E*). Although CC-3 gradually increased throughout the neuron in CPT-treated neurons, M18-1 null neurons showed clear CC-3 activation only in the final stage of cell death, in severely degenerated neurons (Fig. 4*E*). Thus, in contrast to typical apoptosis, CC-3 is activated later, in the final phase of cell death, on depletion of M18-1 and stx-1.

### Conserved atypical cell death after loss of stx-1 in human neurons

**Figure 5.**
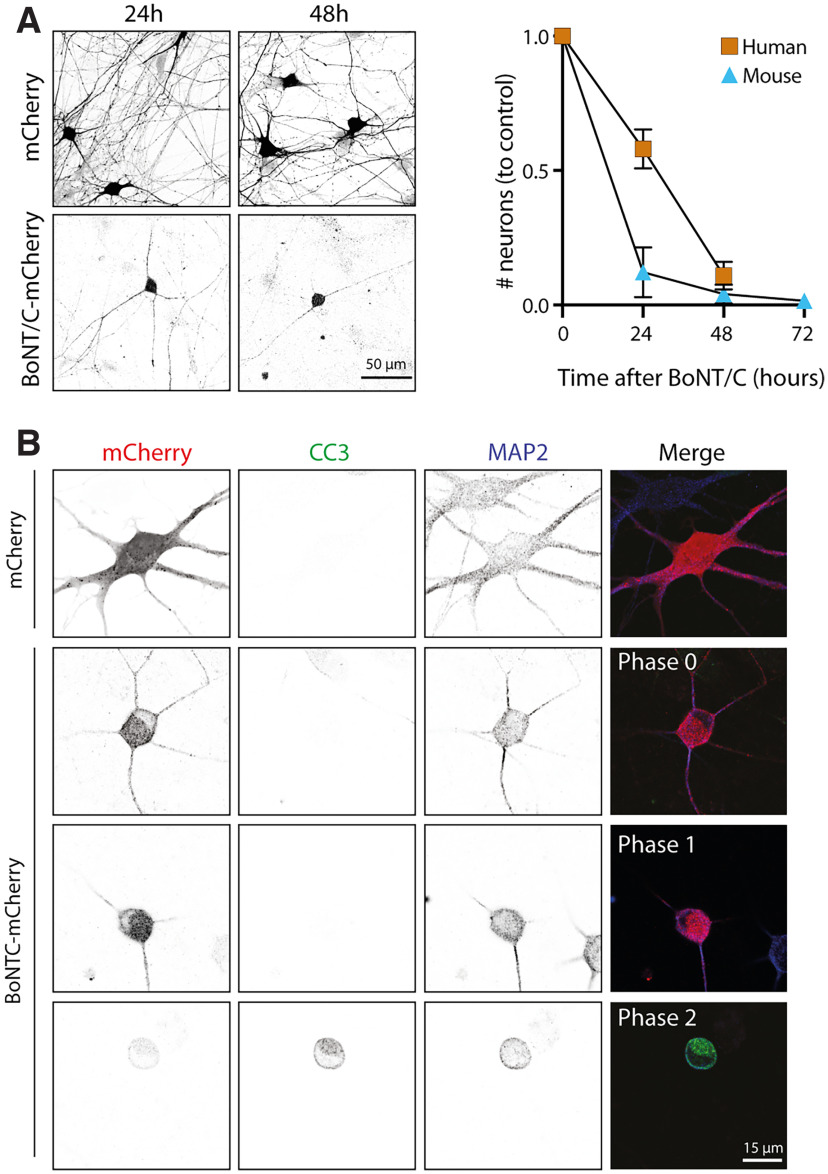
Cell death, morphologic stages, and late cleaved caspase 3 activation are conserved in human neurons treated with BoNT/C. ***A***, Human neurons treated with BoNT/C-mCherry die within 48 h (human neurons, *n*/*N* = 70 FoV/2 cultures; mouse neurons, *n*/*N* = 60 FoV/2 cultures). Scale bar, 50 μm. ***B***, Typical examples of human neurons treated with mCherry or BoNT/C-mCherry and stained for mCherry, CC-3, and MAP2. Scale bar, 15 μm.

To investigate whether this route of cell death is conserved in human neurons, stx-1 was depleted in human iPSC-derived neurons. Similar to mouse neurons, human neurons died after Stx-1 depletion, albeit at a slightly slower rate (Fig. 5*A*). Moreover, human neurons showed similar morphologic phases of neurite loss before cell death on stx-1 loss (Fig. 5*B*). Immunofluorescent staining for CC-3 in Stx-1 depleted human neurons revealed that also in human neurons, CC-3 was only activated in the final stage of cell death in severely degenerated neurons (Fig. 5*B*). Together, these data show that the atypical cell death after loss of stx-1 characterized by consecutive neurite loss and late CC-3 activation is a shared property between murine and human neurons.

### No effect of apoptosis inhibitors in arresting atypical cell death in M18-1 null or stx-1 depleted neurons

**Figure 6.**
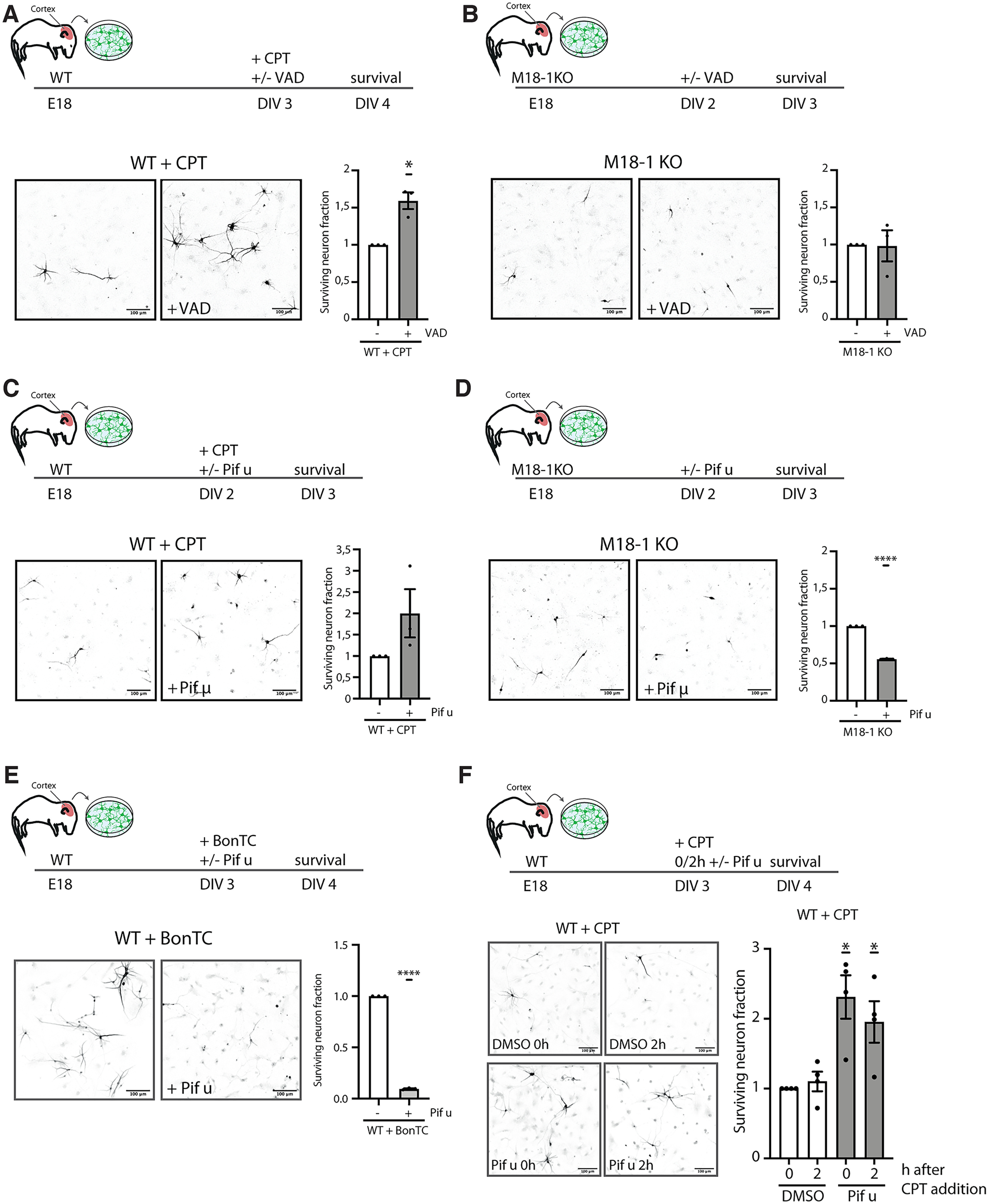
Atypical cell death of M18-1 null or BoNT/C-treated neurons is not driven by classical apoptosis. ***A***, Typical example and quantification of neuronal survival of WT neurons treated with CPT or CPT in combination with Z-VAD-fmk for 24 h at DIV 3. Mean + SEM of three independent experiments (*p < 0.05, 1-sided *t* test). ***B***, Typical example and quantification of neuronal survival of M18-1 null neurons at DIV 3 and M18-1 null neurons treated with Z-VAD-fmk for 24 h. Mean + SEM of three independent experiments. ***C***, Typical example and quantification of neuronal survival at DIV 3 of WT neurons treated with CPT or CPT in combination with pifithrin-μ for 24 h. Mean + SEM of three independent experiments. ***D***, Typical example and quantification of neuronal survival at DIV 3 of M18-1 null neurons and M18-1 null neurons treated with pifithrin-μ for 24 h. Mean + SEM of three independent experiments (****p < 0.0001, 1-sided *t* test). ***E***, Typical example and quantification of neuronal survival of WT neurons transfected with BonTC-mCherry RNA in combination with DMSO or pifithrin-μ for 24 h. Mean + SEM of three independent experiments (****p < 0.0001, 1-sided *t* test). ***F***, Neuronal survival after direct or 2 h delayed pifithrin-μ addition. Mean + SEM of four independent experiments (*p < 0.05, 1-sided *t* test).

Although caspase activation occurred later in M18-1 null neurons than during typical apoptosis, we tested whether cell death could still be arrested, as in the case of typical apoptosis, by blocking caspase activation (Fricker et al., 2018) or inhibition of p53 activation (Strom et al., 2006). Neurons were treated with general caspase inhibitor Z-VAD-fmk, or the p53 inhibitor pifithrin-μ (Fig. 6). As expected, treatment with Z-VAD-fmk rescued apoptotic cell death induced by CPT in WT neurons (Fig. 6*A*). In contrast, Z-VAD-fmk treatment did not alter survival in M18-1 null neurons (Fig. 6*B*). Inhibition of p53-mediated apoptosis by addition of pifithrin-μ promoted cell survival in WT neurons treated with CPT both when added directly together with CPT or when added 2 h after CPT treatment (Fig. 6*C*,*F*). However, pifithrin-μ treatment in M18-1 null neurons did not prevent cell death and resulted in even lower viability of these neurons (Fig. 6*D*). Similarly, addition of pifithrin-μ during the more acute stx-1 depletion resulted in reduced neuronal survival compared with control stx-1 depleted neurons (Fig. 6*E*). Together, these data show that blocking caspase activation or p53-mediated apoptosis is insufficient to arrest cell death on M18-1 or stx-1 depletion.

## Discussion

This study characterized neurodegeneration caused by depletion of the neuronal SNARE proteins Stx-1 and M18-1 and compared these characteristics to classical apoptosis induced by camptothecin. Hallmarks of classical apoptosis, like cleaved caspase 3 activation, p53 phosphorylation, and condensed nuclei, were also observed in hippocampi of M18-1 null mice. However, cell death on M18-1 or stx-1 depletion showed distinct features not shared with typical apoptosis in neurons, especially (1) consecutive neurite retraction before the actual cell death occurs; (2) late CC-3 activation, only after neuron disassembly; and (3) no effects of classical apoptosis inhibitors.

Apoptosis is a final common pathway for a multitude of triggers that lead to neuronal cell death, for instance, during excitotoxicity or metabolic stress, for example, during ischemia/stroke (for review, see Fricker et al., 2018). Different upstream signal transduction cascades have been characterized, for instance the intrinsic apoptotic pathway characterized by increased mitochondrial membrane permeability and cytochrome C release, or the extrinsic apoptotic pathway characterized by ligation of cell death receptors and caspase 8 activation (Muzio et al., 1996; Boldin et al., 1996; Varfolomeev et al., 1998; Krajewska et al., 2011). These different apoptotic pathways converge onto the cleavage of CC-3, the executor caspase that once activated is responsible for dismantling the cell (Tewari et al., 1995; Liu et al., 1997; Kothakota et al., 1997; Julien and Wells, 2017). In contrast, on M18-1 or stx-1 depletion, CC-3 is activated only after the cell is almost completely dismantled. This argues against an executive role for CC-3 in cell death on M18-1 or stx-1 depletion. Moreover, although both intrinsic and extrinsic apoptosis were rescued or at least delayed by inhibition of caspase or p53 activity, cell death on M18-1 or stx-1 depletion was not. This is in line with previous observations that axonal breakdown and cytochrome C release from dying neurons treated with BoNT-C holotoxin were not inhibited by caspase inhibitors (Berliocchi et al., 2005). Also, primary cultured M18-1 KO neurons are not beyond the rescue point, as overexpression of the M18-1 homolog M18-3 can rescue neuronal viability in these cultures (Santos et al., 2017). Together, these findings indicate that activated CC-3, in contrast to classical apoptosis, does not execute disintegration in M18-1 or stx-1 depleted neurons. It is plausible that CC-3 activation still induces some hallmarks of apoptosis in dying M18-1 or stx-1 depleted neurons, such as DNA fragmentation, which are inhibited by apoptosis inhibitors (Berliocchi et al., 2005), but these events happen shortly before cell death in the remaining neurite-deprived somata of neurons beyond rescue. Hence, we conclude that M18-1 or stx-1 depletion results in cell death ultimately involving, but not driven by, apoptosis.

In addition to apoptosis, other established cell death pathways failed to explain neuronal cell death on M18-1 or stx-1 depletion. Necrosis is characterized by rupture of the plasma membrane usually caused by a passive process such as tissue trauma. The best-defined form of regulated necrosis is necroptosis, which is linked to neuronal cell death in Alzheimer's disease, triggering widespread neuroinflammation (Jayaraman et al., 2021; Thadathil et al., 2021). Necroptosis is executed through phosphorylation of MLKL, which oligomerizes and drives rupture of the cellular membrane (Fricker et al., 2018). However, ruptured membranes and pMLKL immunoreactivity were not detected in dying M18-1 or stx-1 depleted neurons; hence, we find no indications for involvement of necroptosis. In clinical neurodegeneration, such as in Alzheimer's disease, cell death has also been linked to dysregulation of lysosomal function marked by the presence of LC3-positive vacuoles alongside TAU accumulation (Piras et al., 2016). Lysosomal membrane permeabilization causes acidification, release of proteases (cathepsins), and oxidative stress driving cell death (Lee et al., 2010). However, we did not find LC3-positive puncta in M18-1 depleted neurons, arguing against the presence of dysfunctional lysosomes. Furthermore, we also did not find any indication for pyroptosis, which is driven by caspase 1 activation on cell intrinsic inflammatory signaling and should be prevented by pan caspase inhibition (Fricker et al., 2018). Finally, regulated necrosis can occur via ferroptosis, which is caused and characterized by mitochondrial shrinkage. Based on previous work from our lab and others, we have shown that the mitochondria in M18-1 depleted neurons are morphologically normal (Berliocchi et al., 2005; Santos et al., 2017). Together, these considerations indicate that neuronal cell death on M18-1 or stx-1 depletion does not involve other established cell death pathways and should be considered a novel, typical cell death pathway.

The consecutive neurite retraction preceding cell death in M18-1 or stx-1 depleted neurons could be related to the physiological axonal degeneration observed during development when axons compete for trophic support (pruning). Dismantling of the neurites during pruning depends on local caspase 3 activation in the axon (Simon et al., 2016), unlike neurite retraction on M18-1 or stx-1 depletion (which occurs before caspase 3 activation). In addition, pruning or axonal damage is manifested as simultaneous fragmentation of the neurites (Pemberton et al., 2021; Simon et al., 2016), rather than the active, consecutive retraction on M18-1 or stx-1 depletion. Hence, neurite loss during pruning and on M18-1 or stx-1 depletion are two different phenomena, with distinct features. Neurite loss can also be driven by Wallerian degeneration, which occurs following axonal injury. In line with a previous study that found no rescue of neuronal survival on stx-1 depletion in the Wallerian degeneration slow (*Wld*) mice (Berliocchi et al., 2005), we find no indication for Wallerian degeneration in M18-1 or stx-1 depleted neurons.

**Figure 7.**
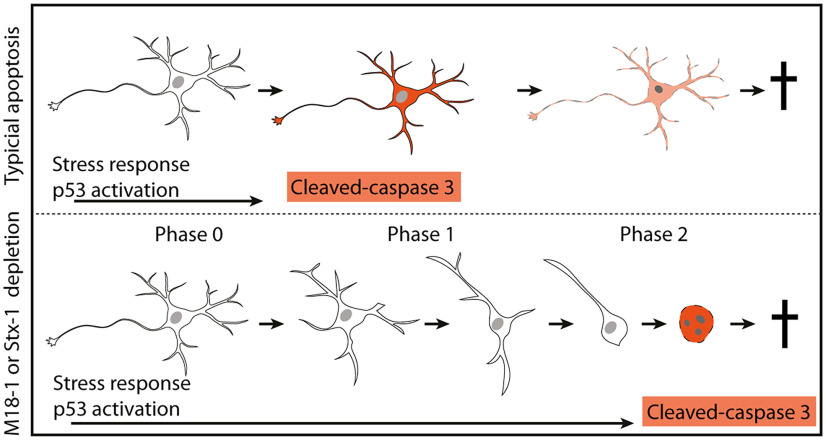
Schematic representation of the atypical neuronal cell death response in absence of M18-1 or stx-1. The atypical route to cell death is characterized by different phases that present progressive neurite loss. Cleaved caspase 3 activity is only apparent after full disruption of the cell. In contrast, classical apoptosis as induced by CPT in WT neurons is hallmarked by the p53-driven apoptotic cascade causing pan cellular accumulation of cleaved caspase 3, which is responsible for the loss of cellular integrity.

The process of consecutive neurite retraction indicates that an upstream dismantling process is activated before actual cell death occurs. Although this process could drive neuronal cell death, it could also act as a protective response aimed at maintaining neuronal viability for as long as possible in unfavorable conditions. Interestingly, inhibition of the major stress reactive protein p53 increased cell death in M18-1 or stx-1 depleted neurons, opposite to the effects on classical apoptosis (Fig. 6). The transcription factor p53 can drive expression of apoptosis-related genes when a cell encounters stress, but p53 activation has also been shown to promote neurite outgrowth and regeneration via a specific set of target genes (Ma et al., 2011; Tedeschi and Di Giovanni, 2009). p53 might be activated to promote neuronal survival under the stress of M18-1 or stx-1 depletion. The presence of activated p53, but not cleaved caspase 3, in the cortex where cell death is known to start only later (Bouwman et al., 2004; Verhage et al., 2000) suggests that p53 activation is not merely a consequence of dying cells in that brain area but instead is triggered by an upstream event. Together, these observations suggest an atypical route to cell death in neurons depleted for the neuronal SNAREs, characterized by two major stages (Fig. 7), (1) an active phase of neurite retraction, resulting in an intact soma and nucleus, with only 1 or 2 short neurites, and (2) a final phase where the soma and nucleus are fragmented, accompanied by caspase 3 activation (Fig. 7).

Neuronal degeneration is not only observed on depletion of MUNC18-1 and syntaxin-1, but also on SNAP25 loss (Delgado-Martínez et al., 2007). Hence, it is plausible that SNAP25 KO neurons demonstrate similar key phenotypes, such as the neurite retraction and late caspase-3 activation, before degeneration. However, some striking differences between degeneration phenotypes might indicate different mechanisms. Degeneration kinetics are slower on SNAP25 depletion; KO neurons survive for at least 1 week and a fraction beyond 14 d (Santos et al., 2017). It has further been reported that high-density cultures of SNAP25 KO neurons survive, which has not been shown for M18-1 or syntaxin-1 depletion (Bronk et al., 2007; Alten et al., 2021). Finally, onset and progression of SNAP25-dependent degeneration differs considerably between neuronal cell types (Hoerder-Suabedissen et al., 2019).

The list of pathways to neuronal cell death is expanding as mechanistic studies on the developing nervous system as well as neurodegenerative diseases provide new insights and also reveals cross talk between different cell death pathways (for review, see Fricker et al., 2018). The current study adds one additional pathway of atypical, staged cell death of neurons on depletion of M18-1 or syntaxin-1 that does not appear to resemble any of the established cell death mechanisms in neurons.
